# Fetus in fetu: a case report

**DOI:** 10.1186/1752-1947-2-2

**Published:** 2008-01-10

**Authors:** Nisreen M Khalifa, Doaa W Maximous, Alaa A Abd-Elsayed

**Affiliations:** 1 Department of Pediatric Oncology, South Egypt Cancer Institute, Assiut University, Assiut, Egypt; 2 Department of Surgical Oncology, South Egypt Cancer Institute, Assiut University, Assiut, Egypt; 3 Department of Public Health and Community Medicine, Faculty of Medicine, Assiut University, Assiut, Egypt

## Abstract

**Introduction:**

Fetus in fetu is a rare abnormality secondary to the abnormal embryogenesis in a diamniotic, monochorionic pregnancy. It is a rare pathological condition and fewer than 100 cases have been reported in the literature.

**Case presentation:**

A 2 month old girl with an abdominal mass since birth, was referred to the Cancer Institute with a suspected diagnosis of a Wilms' tumor. Conventional radiograph of the abdomen revealed a mass containing numerous calcifications. CT scan showed a heterogeneous retroperitoneal mass containing well-defined calcified structures. The decision was made to recommend surgical exploration and the mass was successfully excised. Physical examination of the mass with review of literature confirmed the diagnosis of fetus in fetu.

**Conclusion:**

Although it is a rare condition imaging may play an important role in the correct prospective diagnosis of fetus in fetu. Surgical excision is the recommended treatment.

## Introduction

To our knowledge fetus in fetu was originally described by Meckel in the late 18^th ^century [1]. Fetus in fetu, a term quoted by Willis [2], was first described as a rare condition in which a malformed parasitic twin was found inside the body of its partner usually in the abdominal cavity. It represents an aberration of monozygotic diamniotic twinning in which unequal division of the totipotent inner cell mass of the developing blastocyst leads to the inclusion of a smaller cell mass within a maturing sister embryo.

This pathology is rare and the incidence is 1 per 500 000 births [3], with fewer than 100 reported cases worldwide [4]. The majority of cases occur in infancy, with the oldest reported case occurring in a 47-year-old man [1].

Tharkral et al [5] reported equal male and female prevalence. In 70% of cases, the chief presenting complaint was an abdominal mass [6]. The mass was predominantly retroperitoneal in 80% of cases [2], while reported uncommon sites include the oral cavity [1], sacrococcygeal region [7], and scrotum [4].

## Case presentation

A two month old girl was hospitalized because of an abdominal mass present since birth. On physical examination, a smooth, firm, nontender, right flank mass was present.

**Figure 1:**
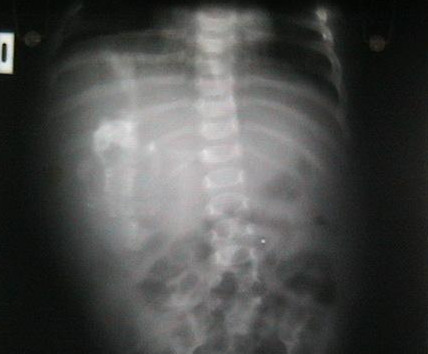
Anteroposterior abdominal radiograph demonstrates a soft tissue mass in the right hemiabdomen. The mass contains calcified osseous-appearing structures of varying sizes and shapes (see arrows)

**Figure 2:**
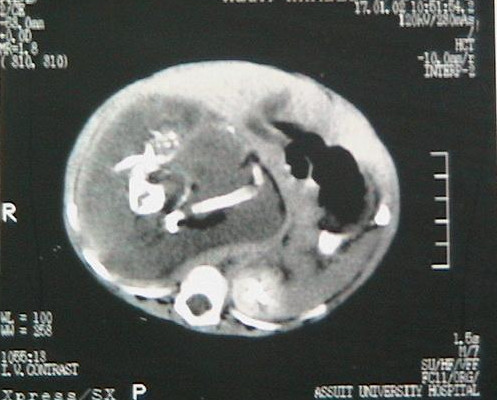
Computed Tomography scan of the patient's abdomen reveals a large retroperitoneal soft tissue mass. There are long hyperdense opacities that resemble fetal bones (see arrows)

Conventional abdominal radiography showed a large right abdominal mass, which was predominantly soft tissue containing several calcifications [Figure 1]. Abdominal ultrasonography revealed a large, hyperechoic, heterogenous intra-abdominal mass that appeared to contain areas of calcification. CT scan didn't add much as regard to the origin of the mass or its nature [Figure 2], so the decision was made to recommend surgical exploration.

Elective laparotomy was performed. We found a large retroperitoneal cystic mass attached to the lower pole of the right kidney pushing the hepatic flexure of the colon anteriorly. The sac contained a clear fluid and the fetus within had grossly visible limbs. The cystic component was decompressed and the mass dissected off the retroperitoneum with ligation of the feeding vessels. The baby did well and was discharged on the 6^th ^day postoperatively.

**Figure 3:**
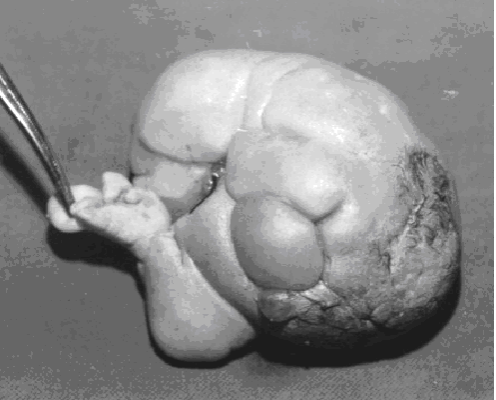
The postoperative specimen shows a fairly well developed fetus lying on its back, the photograph demonstrates its rudimentary digits.

Macroscopic examination revealed a soft tissue mass resembling a fetus, attached to the membranous sac via a 20 mm cord like structure. The mass weighed 250 gm, and measured 12 × 10 × 8 cm. It was covered entirely with intact skin, there were 2 malformed lower limbs, the right measured 6 cm long, with a 4.6 cm foot, the left measured 6 cm long with a 3.5 cm foot. There were 4 rudimentary digits with a big toe in the right foot., there was also a rudimentary upper limb measuring 5 cm long. [Figure 3]. Microscopic examination of the mass was performed and revealed the presence of lungs, liver and spleen.

After four years our patient has shown no recurrence or complications and is leading a completely normal healthy life.

## Discussion

Fetus in fetu occurs most commonly in the upper retroperitoneum, while teratoma usually occurs in the lower abdomen, ovaries, or sacrococcygeal region [5]. In this case our patient's mass was in the retroperitoneum.

The fetus was always anencephalic. The vertebral column and the limbs were present in the fetus in fetu in almost all cases [91% and 82.5%, respectively]. There is controversy as to whether fetus in fetu is a distinct entity or represents a highly organized teratoma. A teratoma is defined as a neoplasm with a slight potential for malignancy that is composed of multiple tissues foreign to the part in which they are located [8]. It often is difficult to make a distinction between teratomas and vestigial remnants that result from abortive attempts at twinning. As a result, some authors think that fetus in fetu is within the spectrum of abnormalities that can result from the anomalous embryogenesis in a diamniotic monochorionic pregnancy. The spectrum includes conjoined symmetric twins; parasitic fetuses; embryonic vestigial fetal inclusions; and organized teratoma, Thus, some authors claim that fetus in fetu is only a well-differentiated highly organized teratoma [8].

However, many other investigators suggest that fetus in fetu is a pathologic entity that is distinct from teratoma for several reasons [3]; Malignant degeneration associated with fetus in fetu is extremely rare, with only one reported case to our knowledge [3].

A final important feature that has been used to distinguish between fetus in fetu and teratoma is the presence of a vertebral column. Willis [2] emphasized that the identification of a vertebral column secures the diagnosis of fetus in fetu and differentiates this entity from teratoma. Identification of the vertebral column indicates that fetal development of the included twin must have advanced at least to the primitive streak stage to develop a notochord, which is the precursor of the vertebral column [2]. Occasional cases have been reported in which the spinal column could not be identified at imaging [6]. These cases probably were due to an underdeveloped and markedly dysplastic spinal column that prevented the identification of vertebral bodies at imaging [9]. The intraabdominal fetus in fetu is usually contained in a complete sac, without any major vascular connections to the host, which was the situation in our reported case.

In case reports in the medical literature up to 1980 preoperative diagnosis of fetus in fetu was made in only 16.7% of cases because CT scan was not performed. Since that time CT scan has proven very helpful in suggesting the preoperative diagnosis [10]. The differential radiological diagnoses are teratoma and meconium pseudocyst. Indeed, these masses often have calcified components, so they were sometimes difficult to differentiate with fetus in fetu [7].

In our case imaging did not play an important role in our ability to make a preoperative diagnosis.

## Conclusion

Fetus in fetu is a rare and interesting entity that typically presents in infancy or early childhood. Current imaging modalities may allow us to accurately diagnose the condition before surgery. Complete excision is curative and allows confirmation of the diagnosis.

## Competing interests

The author(s) declare that they have no competing interests.

## Authors' contributions

NMK: Examined the patient, admitted her, ordered the investigations and then referred her to the surgery department and participated in writing the drafts, DWM Carried out the surgery, AAA-E: followed up the patient's condition and survival after surgery, wrote the drafts and the final manuscript and kept track of the patient records and investigations. All authors read and approved the final manuscript.

## Consent

Written informed consent was obtained from the parents of our patient for publication of this Case report and any accompanying images. A copy of the written consent is available for review by the Editor-in-Chief of this journal.
